# Individual, social and national coping resources and their relationships with mental health and anxiety: A comparative study in Israel, Italy, Spain, and the Netherlands during the Coronavirus pandemic

**DOI:** 10.1177/1757975921992957

**Published:** 2021-02-18

**Authors:** Adi Mana, Sabina Super, Claudia Sardu, Dolors Juvinya Canal, Neuman Moran, Shifra Sagy

**Affiliations:** 1 School of Behavioral Sciences, Peres Academic Center, Israel; 2 Health and Society Group, Wageningen University, Netherlands; 3 The Department of Medical Sciences and Public Health of the University of Cagliari, Italy; 4 Health Promotion Chair, Research Group Health and Healthcare, University of Girona, Spain; 5 The Department of Education, Ben-Gurion University of the Negev, Beer Sheva, Israel; 6 Martin Springer Center for Conflict Studies, Ben-Gurion University of the Negev, Beer Sheva, Israel

**Keywords:** Social support, stress, sense of coherence, sense of national coherence, trust, COVID-19, mental health, anxiety

## Abstract

Employing the salutogenic model, we asked how individuals in different countries cope with the COVID-19 crisis and stay healthy. We were interested in exploring the individual (i.e. sense of coherence) as well as the social and national resources (i.e. social support, sense of national coherence, and trust in governmental institutions) that could explain levels of mental health and anxiety during the outbreak of the pandemic. Data collection was conducted via convenience sampling on online platforms, during the end of March and the beginning of April 2020. The data included four samples: 640 Israeli participants (319 males), 622 Dutch participants (177 males), 924 Italian participants (338 males) and 489 Spanish participants (117 males); age range of 18–88 years. The questionnaires included standard tools (MHC-SF, GAD-7, SOC, SONC). Several questions were adapted to the context of coronavirus and measured levels of exposure to COVID-19, trust in governmental institutions, and social support. The results significantly confirmed the suggested salutogenic model regarding the contribution to individual and national coping resources to anxiety levels and mental health. The patterns of the coping resources in explaining anxiety and mental health were similar in the four samples, and SOC was the main predictor these outcomes. Despite these similarities, a different pattern and also different magnitudes of the predictive value of the coping resources were found for the two different reactions: anxiety vs. mental health. While SOC and situational factors (like financial threat) were significant in explaining anxiety levels, the SOC and national resources were found as significant in explaining mental health levels. The findings support the salutogenic approach in studying reactions during pandemic time. They also shed some light on the difference between pathogenic and salutogenic measures in studying psychological reactions to stressful situations.

## Introduction

The COVID-19 pandemic has affected the lives of millions worldwide, causing great uncertainty and anxiety. In addition to severe physical health consequences, the pandemic is also having an impact on the general population in terms of mental health and well-being (1). Former studies have indicated that the virus's spread over a given territory or the level of actual health event were not the only factors that predicted mental health and anxiety levels (2). Moreover, differences in the responses to the pandemic could be observed between and within countries, as individuals and nations differ in their resources to cope with such a crisis (3). Thus, it seems valuable to study which resources play a role in coping with a pandemic in a health-promoting way and to compare the pattern of these resources in promoting mental health and reducing anxiety levels across countries.

Our study employs the salutogenic approach (4) that, in contrast to pathogenesis, focuses on the study of the origins of health, illuminates salutary factors that promote health, reduces distress reactions, and explains successful coping with stress (5).

While the COVID-19 pandemic is a global phenomenon, it appears that each nation has developed its own way of managing it (1,6). Thus, the first aim of our study was to compare levels of mental health and anxiety in four countries that differ in their situation and management of the pandemic in its first stage. Both measures are commonly used as indicators of psycho-physiological distress. However, anxiety is usually considered a pathological construct while mental health is a salutogenic one (7). Our second purpose relates to the differential patterns of coping resources in explaining these two measures.

### Emotional reactions

Various psychological vulnerability factors may play a role in anxiety levels, including individual differences of intolerance of uncertainty, perceived vulnerability to disease, and anxiety proneness (3). We hypothesized that general anxiety will be explained mainly by state related variables, such as level of health or financial threat due to the pandemic situation (8).

Our second measure of mental health refers to a subjective evaluation of one’s state of wellbeing (7). It is not merely the absence of anxiety, but it also relates to the presence of positive feelings, positive functioning in individual and community life, and life satisfaction (9). We expected the level of mental health to be explained less by the situational variables of the pandemic risk and more by one's coping resources (8,9).

### Coping resources as explanatory factors

Our main research question relates to the influence of individual, social, and national coping resources on the emotional reactions to acute stress situations.

On the individual level we included the sense of coherence (SOC) (4), as a core salutogenic construct. SOC is defined as a global orientation in life which enables people to view life as comprehensible, manageable, and meaningful (4). People with a stronger SOC are better able to understand the stressor (comprehensibility), are better capable to select an appropriate strategy and available resources to deal with the stressor (manageability), and have a stronger feeling that engaging with the stressor is a meaningful process (meaningfulness). A strong SOC has been found in research to be associated with better quality of life, reduced anxiety, and better mental health (4,5).

However, when people face collective stressors, the resources of the group are also crucial (4). Therefore, we also explored social and national coping resources. Social support was found to act as a buffer against adverse life events and to support mental health in times of crisis. Social support and SOC were found to have significant independent and shared contributions to explained variance on the mental health index (10).

On a national level various coping resources are available that allow individuals and communities to cope well under stress. In this study we focus on two resources: the sense of national coherence (SONC) and trust in governmental institutions. The salutogenic concept of SONC (11) reflects an enduring tendency to perceive one's national group as comprehensible, meaningful, and manageable. Strong SONC was found to be an important factor for resilience especially in conflict areas (12).

Similarly, trust in governmental institutions can mitigate the impact of a pandemic on mental health, as has been suggested previously during the COVID-19 epidemic (3, 6). Loss of trust in the aftermath of a disaster was found as a potential factor in worsening health problems (13).

### The context of the study

Our study investigated these measures across four different countries: Israel, the Netherlands, Italy, and Spain. In each country, the pandemic situation was different during the period of data collection. Based on the database of OurWorldInData (14), during that period (19 March–24 April 2020), Israel was significantly lower as compared to the Netherlands, Italy, and Spain in the spread and the damage of the pandemic. The strategy of the governments to deal with the pandemic was also different at this stage (14,15,16). In Israel, Italy, and Spain the government imposed a complete lockdown. Schools and commercial activities with the exception of essential ones were closed, and restrictions were imposed on leaving home, except for certain justified reasons. In the Netherlands, however, the national measures were implemented in what the government called ‘an intelligent lockdown’, allowing most shops to remain open and allowing people to go outside for recreational purposes (17). Moreover, a range of emergency financial schemes for employers and self-employed people were in place, while in Israel, Italy, and Spain the governmental financial support during this period was not clear. On this background, we expected to find different levels of reactions and patterns of coping resources in the four samples as follows.

***The study hypotheses*:**

1. Depending on the pandemic situation, levels of the reactions will be different among the four countries. Higher levels of anxiety and lower levels of mental health were expected in Italy and Spain than in the Netherlands and Israel.
2. Based on the salutogenic approach, a similar pattern of coping resources was expected to explain levels of anxiety and mental health in the four countries.
3. Different patterns of factors, however, were expected to explain mental health vs. anxiety. Mental health would be explained more by personal, social, and national coping resources, while anxiety response would be explained more by the situational risk factors.

## Method

### Participants

Data collection took place from 19 March–24 April 2020. Recruitment of participants was conducted via an online survey platform and social media networks. The current data analysis included four samples: participants from Israel, the Netherlands, Italy, and Spain (the following data and results will be presented according to this order): 619 Israelis (303 males, 48.9 %), with an age range of 18–75 years (mean = 38.61, SD = 13.11); 622 Dutch (177 males, 29.3 %), with an age range of 19–88 (mean = 44.71, SD= 18.02); 924 Italians (338 males, 36.6 %), with an age range of 18–86 (mean = 41.67, SD = 16.84); and 489 Spanish (117 males, 23.9 %), with an age range of 18–80 (mean = 48.32, SD = 13.86).

Very few participants in the four samples reported that they had been diagnosed with Coronavirus (3 (0.5%), 1 (0.2%), 4 (0.4%), 25 (5.1%)). About a quarter of the participants reported that they are in a high-risk group because of their age or health status 110 (17.8%), 155 (25.6%), 198 (21.4%) and 129 (26.4%). Some of the participants reported that they were or had been in quarantine 53 (8.6%), 70 (11.6%), 294 (31.8%) and 94 (19.2%). Most of the Israeli and Italian participants estimated that they would suffer financially from COVID-19 crisis 466 (75.2%), 563 (60.9%), while smaller numbers estimated so in Spain 204 (41.7%), and only 68 (11.2%) of the Netherlands participants estimated that they would suffer financially.

### Instruments

The study instrument comprised structured and self-reported questionnaires that were back translated (18) from English to Hebrew, Dutch, Italian, and Spanish.

#### The Generalized Anxiety Disorder (GAD-7, Spitzer, Kroenke, and Löwe, 2006)

The seven items of this scale enquired about the degree to which the participant has been bothered by feeling nervous, anxious, worried, restless, annoyed, and afraid during the two weeks prior to answering the questionnaire. Each item was scored on a four-point Likert scale (0–3), with total scores ranging from 0 to 21 where higher scores reflect greater severity of anxiety. Internal consistency of the questionnaire was estimated at 0.89 (19) and in the current study α = 0.91, 0.85, 0.88, 0.90.

#### Mental health continuum (MHC-SF, Lamers, Westerhof, Bohlmeijer, ten Klooster, and Keyes, 2011)

The scale includes 14 items measuring the three components of well-being: emotional, social, and psychological. The questionnaire was adapted to the current context and based on the experiences the participants had over the last two weeks (never, once in these two weeks, about once a week, two or three times a week, almost every day, or every day). Internal consistency of the questionnaire was estimated at 0.89 (7) and in the current study α = 0.90, 0.89, 0.91, 0.94.

#### Sense of coherence (SOC-13, Antonovsky, 1987)

The 13 items, on a seven-point Likert scale, explore the participants’ perceptions of the world as comprehensible, meaningful, and manageable. The Italian SOC version was distributed to the Italian sample (20). The α values in former studies using SOC-13 range from 0.70 to 0.92 (21) and in this study the α = 0.79, 0.85, 0.81, 0.82.

#### Sense of national coherence (SONC, Mana, Srour, and Sagy, 2019)

The eight items on a seven-point Likert scale (1 = totally agree, 7 = totally disagree) explore the participants’ perceptions of his/her own society as comprehensible, meaningful, and manageable. Internal consistency of the questionnaire was estimated at 0.80 (11) and in the current study α = 0.84, 0.70, 0.77, 0.81.

#### Trust in governmental and other institutions

A seven-item questionnaire regarded level of trust in relevant institutions (i.e. media, prime minister, police, government, ministry of finance, ministry of health, health-care workers, and hospitals) on a five-point Likert scale (1 = very much, 5 = not at all). Internal consistency was α = 0.77, 0.85, 0.85, 0.86.

#### Social support

Three items explored feelings of support that the participant feels he/she receives from family members, from the community in the neighbourhood or settlement, and from virtual communities (i.e. social networks, Twitter, Facebook), on a five-point Likert scale (1 = very much, 5 = not at all).

#### Socio-demographic variables

Demographic information (gender, age, marital status) was collected.

#### Level of risk and exposure to COVID-19

We explored both health and financial risk by asking if the participant: 1) was part of a risk group because of his/her age and/or medical condition; 2) had been in quarantine; and 3) had been diagnosed with COVID-19. We also explored the participant’s estimation of financial risk: To what extent do you think you will suffer financially from the Coronavirus crisis? (1 = very low, 5 = very high).

#### Procedure

Prior to data collection, we obtained approval from the ethics committees of the participating institution in each county. In Israel, the data were collected via a nonprobability, general population panel (Midgam panel) and in the other countries we used a nonprobability snowball sampling via social media networks (using Qualtrics or other online tools). To reduce the sample selection problem, the invitation letter was distributed among a large variety of social networks and the participants were asked to help in further distributing the link to the questionnaire. In this letter we explained that the research objective was to understand the participant’s experience during the period of Coronavirus. The anonymity of the participants was guaranteed, and no identifying data were collected in the questionnaire. Data were analyzed using SPSS Statistics. Descriptive data were compared using ANOVA. Separated hierarchical regressions were calculated to examine the contribution of coping resources to mental health and anxiety.

## Results

### Preliminary analysis

An ANOVA tested the differences in the levels of the research variables (mental health, anxiety, SOC, SONC, trust, and social support of family, neighbourhood, and virtual community) between the four research groups (Israel, the Netherlands, Italy, and Spain – the results will be presented in that order). As the assumption of homogeneity of variance was not met, we used the Welch’s adjusted F ratio, which was significant at the .01 alpha levels for mental health, .005 for anxiety, and at the .001 alpha for the other variables (see Table 1). Games-Howell Post hoc tests revealed that the levels of SOC, SONC, and trust among the Dutch participants were significantly higher as compared to the other participants, while levels of trust and SONC were the lowest among the Spanish participants. However, levels of family, community, and virtual community were higher among the Spanish participants as compared to the others. As for our first hypothesis, the results revealed that the Dutch participants, as predicted, reported lower levels for anxiety and higher level of mental health as compared to the Italian and the Spanish participants. However, no significant differences in level of anxiety were found between the Israeli participants and the other three samples, and their level of mental health was lower compared to the Netherlands participants. Therefore, the first hypothesis was not confirmed.

**Table 1.** Means, standard deviations and one-way analyses of variance in mental health, anxiety and coping resources in Israel, the Netherlands, Italy and Spain.

*Measure*	*Israel*		*The Netherlands*		*Italy*		*Spain*		*Sig F*	
	M	SD	M	SD	M	SD	M	SD		
**Mental health**	3.95	1.05	4.40	0.87	4.04	0.99	4.18	1.12	.010	3.934
**Anxiety**	7.46	5.65	5.57	3.79	8.29	4.96	7.72	5.17	.005	4.776
**SOC**	4.54	0.84	4.97	0.90	4.41	0.95	4.80	0.91	.000	7.613
**SONC**	4.08	1.16	4.61	0.75	3.55	1.03	3.22	1.09	.000	141.887
**Trust**	3.24	0.78	3.89	0.52	3.89	0.75	2.53	0.78	.000	233.758
**Family support**	4.23	1.05	4.07	0.92	4.08	1.01	4.65	0.64	.000	34.995
**Community Support**	3.05	1.37	2.86	1.22	2.80	1.19	3.72	1.20	.000	45.896
**Virtual Support**	3.32	1.32	3.06	1.33	2.91	1.28	3.60	1.26	.000	27.684

#### Hierarchical regression

Separate hierarchical regressions were calculated to test the second and the third hypotheses. Demographic variables of age and dummy variables of belonging to a risk group, financial risk, and being in quarantine were controlled for in the model. In the first step, control variables were entered. In the second step, SOC, SONC, trust, and social support were added. Table 2 shows the final step of the regression model.

**Table 2.** A summary of the hierarchical regression analysis between health and economic risk factors, gender, SOC, SONC, trust and social support mental health and anxiety.

*Mental Health*																
*Country*	*Israel*				*The Netherlands*				*Italy*				*Spain*			
Variables	B	SE B	β	t	B	SE B	β	t	B	SE B	β	t	B	SE B	β	t
Model 1																
**Gender**	−0.02	0.09	−0.01	−0.18	0.07	0.08	0.04	0.83	0.12	0.08	0.06	1.51	−0.08	0.13	−0.03	−0.64
**Health risk group**	−0.18	0.13	−0.07	−1.46	−0.21	0.10	−0.11	−2.14	−0.05	0.10	−0.02	−0.55	−0.18	0.13	−0.07	−1.39
**Quarantine**	0.11	0.16	0.03	0.66	−0.19	0.11	−0.07	−1.73	−0.09	0.08	−0.04	−1.08	0.15	0.14	0.05	1.07
**Age**	0.01	0.00	0.15	3.14	0.02	0.00	0.31	6.02	0.02	0.00	0.24	5.69	−0.00	0.00	−0.07	−1.31
**Economic risk**	−0.14	0.04	−0.16	−3.72	−0.17	0.05	−0.15	−3.80	−0.10	0.03	−0.12	−3.23	−0.13	0.05	−0.12	−2.46
Model 2																
**Gender**	0.01	0.07	0.00	0.13	0.06	0.07	0.03	0.91	0.12	0.06	0.06	2.02	0.05	0.11	0.02	0.41
**Health risk group**	−0.01	0.10	−0.01	−0.13	−0.06	0.08	−0.03	−0.77	0.02	0.07	0.01	0.28	−0.19	0.11	−0.07	−1.71
**Quarantine**	0.18	0.13	0.05	1.45	−0.02	0.09	−0.01	−0.18	−0.00	0.07	−0.00	−0.03	0.12	0.12	0.04	1.05
**Age**	−0.00	0.00	−0.03	−0.84	0.00	0.00	0.04	0.92	0.01	0.00	0.09	2.65	0.00	0.00	−0.02	−0.45
**Economic risk**	−0.03	0.03	−0.03	−1.00	−0.07	0.04	−0.06	−1.86	0.01	0.02	0.01	0.21	−0.02	0.05	−0.02	−0.51
**SOC**	0.57	0.05	0.46	11.86	0.44	0.04	0.45	11.76	0.49	0.03	0.47	14.31	0.41	0.06	0.33	7.29
**SONC**	0.20	0.03	0.22	5.86	0.12	0.05	0.11	2.65	0.09	0.04	0.10	2.69	0.01	0.05	0.01	0.23
**Trust**	0.02	0.05	0.02	0.49	0.03	0.07	0.02	0.39	0.15	0.05	0.11	3.17	0.23	0.07	0.16	3.40
**Family support**	0.10	0.04	0.10	2.69	0.08	0.03	0.09	2.42	0.13	0.03	0.14	4.32	0.31	0.08	0.18	3.71
**Community support**	0.07	0.03	0.09	2.15	0.10	0.03	0.14	3.55	0.14	0.03	0.17	5.49	0.06	0.04	0.06	1.26
**Virtual Support**	0.05	0.03	0.06	1.48	0.08	0.02	0.13	3.55	0.02	0.02	0.02	0.77	0.15	0.04	0.17	3.88
Anxiety																
Model 1																
**Gender**	−1.73	0.46	−0.15	−3.75	−1.27	0.36	−0.15	−3.55	−1.16	0.40	−0.11	−2.96	−0.72	0.60	−0.06	−1.19
**Health risk group**	0.82	0.65	0.06	1.26	0.49	0.44	0.06	1.11	0.69	0.50	0.06	1.42	−0.66	0.60	−0.07	−1.11
**Quarantine**	−1.21	0.85	−0.06	−1.43	1.20	0.48	0.10	2.51	1.10	0.43	0.10	2.56	−0.66	0.63	0.06	1.18
**Age**	−0.04	0.02	−0.10	−2.17	−0.05	0.01	−0.25	−4.97	−0.02	0.01	−0.07	−1.70	0.01	0.01	0.06	0.90
**Economic risk**	1.30	0.19	0.28	6.73	0.66	0.20	0.13	3.29	0.78	0.15	0.19	5.03	0.92	0.24	0.19	3.82
Model 2																
**Gender**	−1.49	0.41	−0.13	−3.61	−0.91	0.32	−0.11	−2.86	−0.87	0.35	−0.08	−2.47	−0.29	0.54	−0.02	−0.53
**Health risk group**	0.29	0.57	0.02	0.51	0.03	0.40	0.00	0.09	0.46	0.43	0.04	1.09	−0.59	0.53	−0.05	−1.09
**Quarantine**	−1.33	0.74	−0.06	−1.81	0.66	0.43	0.06	1.53	0.44	0.38	0.04	1.15	0.56	0.56	0.04	1.01
**Age**	0.02	0.02	0.05	1.28	−0.01	0.01	−0.04	−0.82	0.02	0.01	0.05	1.37	0.00	0.01	0.02	0.42
**Economic risk**	0.78	0.17	0.17	4.52	0.28	0.18	0.06	1.55	0.43	0.14	0.11	3.09	0.43	0.22	0.09	1.96
**SOC**	−3.48	0.28	−0.52	−12.35	−2.16	0.18	−0.50	−12.00	−2.31	0.20	−0.44	−11.64	−2.74	0.27	−0.48	−10.25
**SONC**	0.02	0.20	0.00	0.10	−0.23	0.22	−0.05	−1.06	0.03	0.20	0.01	0.15	−0.18	0.24	−0.04	−0.77
**Trust**	−0.04	0.29	−0.01	−0.13	0.48	0.32	0.06	1.50	−0.46	0.28	−0.07	−1.65	0.62	0.33	0.10	1.92
**Family support**	−0.16	0.22	−0.03	−0.74	0.04	0.17	0.01	0.22	−0.29	0.18	−0.06	−1.62	−0.44	0.40	−0.06	−1.10
**Community support**	0.30	0.18	0.07	1.69	−0.08	0.13	−0.03	−0.62	−0.44	0.15	−0.11	−2.88	−0.22	0.21	−0.05	−1.02
**Virtual Support**	0.00	0.19	0.00	0.02	0.23	0.11	0.08	2.04	0.28	0.14	0.07	2.08	0.37	0.19	0.09	1.96

Note: Mental health- Model 1 F(5,536) = 4.82, p = .000; F(5,543) = 13.55, p = .000; F(5,649) = 13.42, p =.000; .F(5,402) = 2.35, p <.05. Model 2 R2 change = 0.40; F change = 62.24, p = .000, F (11,530) = 37.64, p =.000; R2 change = 0.40; F change = 82.19, p = .000, F (11,654) = 55.51 p =.000; R2 change = 0.33; F change = 51.46, p = .000, F (11,548) = 37.66 p = .000; R2 change = 0.32; F change = 31.84, p = .000, F(11,402) = 18.93, p =.000.

Anxiety- Model 1 F(5,541) = 13.04, p <.001; F(5,548) = 15.53, p < .001; F(5,654) = 11.25, p < .001; F(5,402) = 4.03, p < .005. Model 2 R2 change = 0.23, F change = 30.41, p < .001, F (11,541) = 24.47 p = .000; R2 change = 0.21; F change = 27.41, p < .001, F (11,548) = 24.07 p = .000; R2 change = 0.23; F change = 35.48, p < .001, F (11,654) = 26.10 p = .000; R2 change = 0.25, F change = 22.91, p < .001, F(11,402) = .14.94, p = .001.

Israel, The Netherlands, Italy, and Spain accordingly.

**Mental health**. At Step 1, age, gender, and health and financial risk variables predicted approximately 4%, 11%, 9%, and 3% of the variance in mental health scores in the Israeli, Dutch, Italian, and Spanish samples. At this step, financial risk and age scores were significant predictors among the all the Israeli, Dutch and Italian samples, while in Spain only financial risk was significant. Belonging to a health risk group was a significant predictor only among the Dutch sample.

The inclusion of the coping resources at Step 2 led to a significant increase in the variance accounted for by the model. SOC was a significant predictor among all the samples and it was the main significant predictor. Family support was significant in all the samples, while community support was a significant predictor in the Israeli, Dutch, and Italian samples, and support from the virtual community was significant among Spanish and Dutch participants. Levels of SONC significantly predicted mental health in the Israeli, Dutch, and Italian samples. Gender and age were significant predictors only among the Italian sample and trust was significant among the Italian and Spanish samples. Levels of the overall regression model predicted approximately 44%, 49%, 44%, and 35% of the variance in mental health scores.

**Anxiety**. At Step 1, age, gender, and health and financial risk variables predicted approximately 11%, 13%, 8%, and 4% of the variance in anxiety scores in the Israeli, Dutch, Italian, and Spanish samples. At this step, financial risk was a significant predictor among all the samples. Gender was a significant predictor among Israeli, Dutch, and Italian samples. Age was a significant predictor only among the Israeli and the Dutch samples. Having been in quarantine was a significant predictor only in the Italian and the Dutch samples.

The inclusion of the coping resources at Step 2 led to a significant increase in the variance accounted for by the model. SOC was the main predictor among all the samples. Gender was also a significant predictor among the Israeli, Italian, and Dutch samples. At this step, financial risk was a significant predictor among Israeli, Italian, and Spanish participants. Support of virtual community was significant in the Italian and Dutch samples, and community support was significant only in the Italian sample. The overall regression model predicted approximately 34%, 33%, 31%, and 30% of the variance in general anxiety scores in Israel, the Netherlands, Italy and Spain.

The results confirmed the second hypothesis, as similar pattern of coping resources in explaining mental health and anxiety were found in the four samples. However, the third hypothesis was only partly confirmed. In all the samples, the coping resources better explained mental health as compared to anxiety, and the situational factors better explained anxiety than mental health, however, SOC explained anxiety levels better than situational factors.

## Discussion

The study employed a salutogenic perspective and explored the contribution of coping resources to the explanation of anxiety and mental health during the first wave of COVID-19.

First, we found that the levels of emotional responses were quite different in the four samples. As expected, the participants in the Netherlands reported higher mental health scores and lower levels of anxiety as compared to the participants in Italy and Spain. These results can be explained by the lower spread of the pandemic in the Netherlands compared to Italy and Spain. In Israel, however, the levels of anxiety were relatively high, and the levels of mental health were low, despite the low spread of the pandemic at this stage. It seems that the lack of stability of the political and economic systems in Israel, as compared to the Netherlands, could explain the greater vulnerability of the Israeli participants to the global crisis, as compared to the Dutch participants.

As the research samples were not representative, and significantly different in several demographic variables, we must relate to these findings carefully and with strict caution. Nevertheless, the results are consistent with previous studies, indicating that the actual level of virus damage and its spread were not the only factors that predicted stress responses (2, 22). It seems that as COVID-19 was highly covered by the social media all over the world, psychological responses towards the unknown effects of the pandemic appeared among people in different countries, without direct correlation to the levels of the actual risk of infection (22,23). Moreover, psychological responses to a crisis could be related to a variety of factors like the socio-cultural atmosphere of one's community, gender patterns of expressing feelings of fear, and other factors, more than to the ‘objective’ situation of the specific crisis (2,3). The sociological and cultural explanations of these results, however, are beyond the scope of this report.

Our main research question focused on the contribution of coping resources in explaining the participants' levels of mental health and anxiety. The findings mainly confirmed our hypothesis. Coping resources indeed contributed to the predictions of both anxiety and mental health, and SOC was found to be the main predictor of these two reactions. Moreover, as expected, the situational factors (state of health and financial threat) were better predictors of anxiety, while SOC and other coping resources were more dominant in explaining mental health. These findings could be explained by the situational characteristic of the anxiety measure versus the more habitual regular orientation in the life of the mental health measure (8). It appears that one's personal ability to view life as comprehensive, manageable, and meaningful in the chaotic reality of a global pandemic is the most important coping resource in different national and social contexts. This finding supports similar results related to the important role of SOC in the time of COVID-19 (23) and cumulative research from over 30 years that confirms the salutogenic hypothesis (5): a strong sense of coherence (SOC) in the face of hardship enables and advances successful coping and results in less anxiety and better mental health. Understanding the role of coping resources, especially SOC, in promoting mental health during a crisis can lead to a more holistic and salutogenic health care system.

Different patterns were found regarding the predictive value of the national resources for anxiety and mental health: SONC significantly predicted levels of mental health (but not levels of anxiety) in three of the four samples. A previous study revealed that SONC was related to voting patterns (11) and that levels of SONC and mental health were found to be significantly lower among voters for opposition parties (13). It appears that more attention should be paid to the concept of SONC as a potentially significant national resource in coping with the crisis. Despite the global nature of COVID-19, nationality appeared to be a significant factor in dealing with the stressful situation (3). Further research is needed to understand the recent phenomena during the last decade of strengthening national feelings in many countries in the Western world. This seems to be especially significant against the background of a global crisis.

Our study has some limitations that need to be carefully considered. First, the samples are based on a nonprobability convenience sampling. Moreover, the differences found among the four samples in relevant socio-demographic variables limited our ability to conduct accurate comparisons. Therefore, we have no possibility for generalizing the findings on global or national populations. However, internet-based research has many advantages, mainly in terms of timeliness, response rates, and costs (24), and this is especially true in the time of a global pandemic, when the rapid global changes in the pandemic situation required quick responses, and the regulations of social distancing limited the options for other strategies of data collection. Although in the time of the pandemic there was increasing openness and dependency on the internet among a variety of groups, the main challenge of internet-based studies is the non-representability of groups who had no access to an electronic survey. More studies are needed in order to explore the experiences of those specific groups like minorities, immigrants, underprivileged and elderly populations.

Despite these limitations, the findings still point to some theoretical suggestions. First, the similar patterns of coping resources that appeared in such different contexts is very significant and sheds light on the importance of the salutogenic approach in the research of a global pandemic. Understanding the role of coping resources, especially SOC and SONC, in promoting mental health during a crisis can lead to a more holistic and salutogenic health care system. Moreover, based on our findings of the main role of SOC in predicting the mental health of the global population, we suggest exploring SOC in on-going international public health and social surveys.

Second, our findings support the value of a meaningful distinction between the two different responses that were examined in this study: mental health as a salutogenic response and anxiety as a more pathogenic one. We need to understand better, by further research using a mixed methods design, the type of emotional reactions, as well as the patterns of coping resources relevant to study during stressful situations in general, and specifically during a global dramatic pandemic in different contexts.

Understanding the importance of the salutogenic approach view by health systems and leaders, could lead to new directions in health assessment and inform interventions in the pandemic crisis.
